# Clinical trial registration and CONSORT adherence in obstetrics and gynecology journals: a cross-sectional analysis of Chinese- and English-language publications

**DOI:** 10.7717/peerj.21140

**Published:** 2026-04-21

**Authors:** Min Sun, Yongqing Zhu

**Affiliations:** 1 Chinese Journal of Reproduction and Contraception, Shanghai Institute for Biomedical and Pharmaceutical Technologies, Shanghai, China; 2 Reproductive and Developmental Medicine, Obstetrics and Gynecology Hospital of Fudan University, Shanghai, China

**Keywords:** Randomized controlled trial, Clinical trial registration, Journal policy, Instructions for authors, CONSORT statement, Medical journal

## Abstract

**Objective:**

Clinical trial registration and adherence to the Consolidated Standards of Reporting Trials (CONSORT) statement are essential for improving transparency, reducing reporting bias, and enhancing the interpretability of randomized controlled trials (RCTs). However, the extent to which these practices are endorsed by journals and implemented in published trials varies across disciplines and publication contexts. This study aimed to evaluate journal policies regarding clinical trial registration and adherence to the CONSORT statement, and to assess registration and reporting practices among RCTs published in Chinese- and English-language medical journals in the field of obstetrics and gynecology.

**Methods:**

We conducted a cross-sectional analysis of obstetrics and gynecology journals publishing RCTs between 2019 and 2024. Journal instructions for authors were reviewed to determine requirements for clinical trial registration and CONSORT adherence. Published RCTs were assessed for trial registration status and the inclusion of a CONSORT flowchart. Journal characteristics, including membership in the International Committee of Medical Journal Editors (ICMJE) and endorsement of CONSORT, as well as funding support, were extracted. Categorical variables were summarized as frequencies and percentages, and group comparisons were performed using Chi-square tests.

**Results:**

A total of 59 journals (51 English-language and eight Chinese-language) and 1,234 RCTs were included. Among the English-language journals surveyed, 70.6% (36/51) required clinical trial registration and 49.0% (25/51) mandated adherence to the CONSORT statement, compared with 12.5% (1/8) of Chinese-language journals requiring either practice in their journal policies and instructions for authors. Overall, 84.1% (1,038/1,234) of RCTs were registered and 83.5% (1,030/1,234) reported a CONSORT flowchart. Registration and reporting practices were more frequently observed in journals with explicit registration requirements, CONSORT endorsement, ICMJE membership, and funding support.

**Conclusion:**

In this sample of obstetrics and gynecology journals, substantial differences were observed in journal policies and in the implementation of clinical trial registration and CONSORT-related reporting practices between Chinese- and English-language publications. These findings highlight the association between journal policies and reporting transparency, while underscoring the need for continued efforts to strengthen trial registration and reporting standards within the field.

## Introduction

Scientific integrity is fundamental to the advancement of clinical medicine, and randomized controlled trials (RCTs) form the cornerstone of evidence-based healthcare decision-making (DeVito, Bacon & Goldacre, 2020; Higgins et al., 2024). Clinical trial registration represents a pivotal step toward fostering transparency, accountability, and trust in clinical research. However, concerns regarding selective outcome reporting and publication bias have been widely documented (Ranawaka, 2021). A landmark analysis by Turner et al. (2008) demonstrated that a substantial proportion of antidepressant trials registered with regulatory authorities were either unpublished or selectively reported, leading to an overestimation of treatment benefits (Higgins et al., 2024). This practice can lead to an overestimation of the beneficial effects of treatments and underestimation of their harmful effects, resulting in substantial duplication of trials and enormous economic waste (Spector-Bagdady, Ho & Nallamothu, 2019).

Beyond trial registration, adherence to the Consolidated Standards of Reporting Trials (CONSORT) statement has been recognized as a key indicator of reporting quality for RCTs. The CONSORT statement was first published in 1996 to enhance the quality of randomized controlled trial (RCT) reporting (Begg et al., 1996). It was subsequently updated in 2001 (Moher et al., 2001; Schulz et al., 2010). Since its release, CONSORT has played an important role in standardizing the format of reports of randomized controlled trials (RCTs) and improving their overall quality. An increasing number of recognized biomedical journals have endorsed the CONSORT statement, incorporating it into their instructions for authors. In April 2025, three journals—Lancet (Hopewell et al., 2025a), JAMA (Hopewell et al., 2025b), and BMJ (Hopewell et al., 2025c)—simultaneously published the CONSORT 2025 Statement, further updating the reporting standards for RCTs. In this study, we employed the CONSORT flowchart for clinical trials, which has been reported to be associated with improved reporting quality of randomized trials (Egger et al., 2001; Andrade, 2015).

To address these concerns, various international organizations have taken steps to standardize clinical trial processes. In September 2004, the International Committee of Medical Journal Editors (ICMJE) issued a statement that all clinical trials involving human participants should be prospectively registered before they can be considered for publication (Van Heteren et al., 2019). In May 2005, the World Health Assembly supported the ICMJE’s stance and called for the development of a common platform for clinical trial registration (De Angelis et al., 2004). In May 2006, the World Health Organization established the International Clinical Trials Registry Platform (ICTRP) and declared that the “registration of all interventional trials is a scientific, ethical, and moral responsibility”. In October 2008, the Declaration of Helsinki amended its statement to declare that “Every clinical trial must be registered in a publicly accessible database before recruitment of the first subject, and researchers have a duty to make publicly available the results of their research on human subjects” (World Medical Association, 2013).

Despite widespread endorsement of trial registration and the CONSORT statement by journals and international organizations, evidence suggests that compliance with these standards remains variable across medical specialties, regions, and publication contexts. Previous studies have documented gaps between journal policies and actual reporting practices, as well as differences in trial registration rates across countries and disciplines, covering topics such as interpretations of the clinical trial registration management system (Borysowski, Wnukiewicz-Kozłowska & Górski, 2020; Khalil et al., 2005), implementation status (Gray, Gray & Brown, 2017; Azar et al., 2019; Xu & Sun, 2020; Li & Chen, 2018), requirements for clinical trial registration in journal policies or instructions for authors (Wu, Minawaer & Hao, 2018), authenticity of clinical trial data and quality of reporting (Spector-Bagdady, Ho & Nallamothu, 2019; Li & Zhang, 2019; Zhang et al., 2019), and ethical standards (Zhao et al., 2018; Coultas, 2007; Grey et al., 2021). However, much of the existing literature has focused on registration practices in isolation or has examined reporting quality without simultaneously considering journal-level policies, editorial endorsements, and published trial characteristics.

Obstetrics and gynecology constitute a high-volume and clinically impactful research field, with randomized trials directly informing the care of women across the reproductive lifespan. Moreover, reproductive health research frequently faces ethical scrutiny, making the implementation and reporting of trial registration especially relevant. Prior investigations have suggested heterogeneity in trial registration and reporting practices within this specialty (Gupta et al., 2020), highlighting the importance of examining both editorial policies and published trial behavior. Moreover, differences in publication language and journal governance may further influence the adoption and enforcement of trial transparency standards, yet comparative analyses across language contexts remain limited.

Therefore, this study aimed to systematically evaluate clinical trial registration and adherence to CONSORT-related reporting practices in obstetrics and gynecology journals. Specifically, we examined (1) journal policies regarding trial registration and CONSORT endorsement, (2) the prevalence of trial registration and CONSORT flowchart reporting among published RCTs, and (3) the association between journal characteristics and reporting practices in Chinese- and English-language journals. By integrating journal-level policies with article-level reporting behaviors, this study sought to provide a nuanced assessment of trial transparency within the field of obstetrics and gynecology.

## Method

### Study design

We conducted a cross-sectional study to evaluate journal policies on clinical trial registration and adherence to the CONSORT statement, as well as trial registration and reporting practices among RCTs published in obstetrics and gynecology journals.

### Journal selection

Obstetrics and gynecology journals were identified from the Science Citation Index Expanded (SCIE) (https://jcr.clarivate.com/jcr/browse-journals), the “High-quality Scientific and Technical Journals Classification Catalog” issued by China Association for Science and Technology (CAST), and the World Journal Clout Index (WJCI) issued by China National Knowledge Infrastructure (https://wjci.cnki.net/Home/OverseaJournalList?code=004; accessed 5 January 2024). Journals were included if they primarily published research in obstetrics and gynecology and had accessible editorial policies or instructions for authors.

### Investigation of journal requirements regarding clinical trial registration

Membership in the ICMJE (https://web.archive.org/web/20240118051152/https://icmje.org/journals-following-the-icmje-recommendations/) and endorsement of CONSORT were verified using the official ICMJE and CONSORT (https://web.archive.org/web/20230203080815/http://www.consort-statement.org/about-consort/endorsers1) websites by two authors (MS and YZ) on January 5, 2024.

Each journal’s website was reviewed for relevant editorial information, such as journal policy, editorial policy, submission guidelines, or instructions for authors (hereafter referred to as “instructions for authors”) to statistically investigate its requirements for clinical trial registration and whether it requires RCTs to follow the CONSORT statement. Data were independently retrieved and extracted by two authors, and any discrepancies were resolved through a joint review of the authors’ instructions.

### Literature search and eligibility criteria

For the selected English-language journals, a subject search was conducted in the SCIE database using the keyword “randomized controlled trial”. For selected Chinese-language journals, the search was conducted *via* each journal’s official website using the keywords “randomized”, “controlled”, and “clinical trial”. The study period spanned from January 1, 2019 to January 5, 2024, reflecting the most recent five-year publication window to capture current journal policies and reporting practices.

Inclusion criteria were RCTs involving human participants. Exclusion criteria included review articles; editorials, correspondence, conference abstracts; Meta-analyses; Errata, retraction notices; case-control studies, retrospective studies, non-randomized controlled trials, survey studies, registry studies, evaluation studies, articles involving cells, tissues, animals, *etc*.; and articles with unclear methodological descriptions or those that could not be confirmed as RCTs.

### Data extraction

Journal selection, article screening, and data extraction were independently conducted by two authors (MS and YZ). Prior to formal screening, a pilot screening of a subset of articles was performed to ensure consistent application of eligibility criteria. Disagreements were resolved through discussion and consensus.

Extracted variables included journal name, publication language, corresponding author country, trial registration status, trial registry and registration number, funding support, and whether a CONSORT flowchart was reported.

### Statistical analysis

Categorical variables were summarized as frequencies and percentages. Associations between categorical variables were assessed using Chi-square tests. A two-sided *P* value of less than 0.05 was considered statistically significant. Statistical analyses were performed using the IBM SPSS Statistics 23 software (IBM Corp., Armonk, NY, USA).

### Ethical considerations

This study analyzed publicly available journal policies and published articles and did not involve human participants or identifiable personal data. Therefore, institutional review board approval and informed consent were waived.

## Results

Journal characteristics and editorial policies A total of 59 obstetrics and gynecology journals were included, comprising eight Chinese-language journals and 51 English-language journals. Among these, 17 journals were ICMJE members and 21 endorsed the CONSORT statement. Ten journals were members of both (Table 1).

**Table 1:** Requirements for clinical trial registration and CONSORT statement in selected journals.

**Index**	**Total**	**Chinese-language journal**	**English-language journal**
Journals	59	8	51
ICMJE members	17	1	16
CONSORT members	21	0	21
ICMJE & CONSORT members	10	0	10
Trial registration required	37/59 (62.7%)	1/8 (12.5%)	36/51 (70.6%)
CONSORT adherence required	26/59 (44.1%)	25/51 (49.0%)	1/8 (12.5%)

**Notes.**

ICMJE International Committee of Medical Journal Editors CONSORT Consolidated Standards of Reporting Trials

Clinical trial registration was required in the instructions for authors of 70.6% (36/51) of English-language journals and 12.5% (1/8) of Chinese-language journals. Requirements for adherence to the CONSORT statement were stated in 49.0% (25/51) of English-language journals and 12.5% (1/8) of Chinese-language journals (Table 1).

Of the 51 English journals, 16 were ICMJE members, with 14 (87.5%) requiring clinical trial registration in their instructions for authors. In contrast, only 22 (62.7%) of the 35 non-ICMJE member journals required clinical trial registration in their instructions for authors.

Of the 51 English-language journals, 21 endorsed the CONSORT statement, with 13 (61.9%) requiring clinical trial registration according to their instructions for authors. While, only 12 (40.0%) of the 30 journals that did not adhere to the CONSORT statement required clinical trial registration in their instructions for authors.

**Figure 1:**
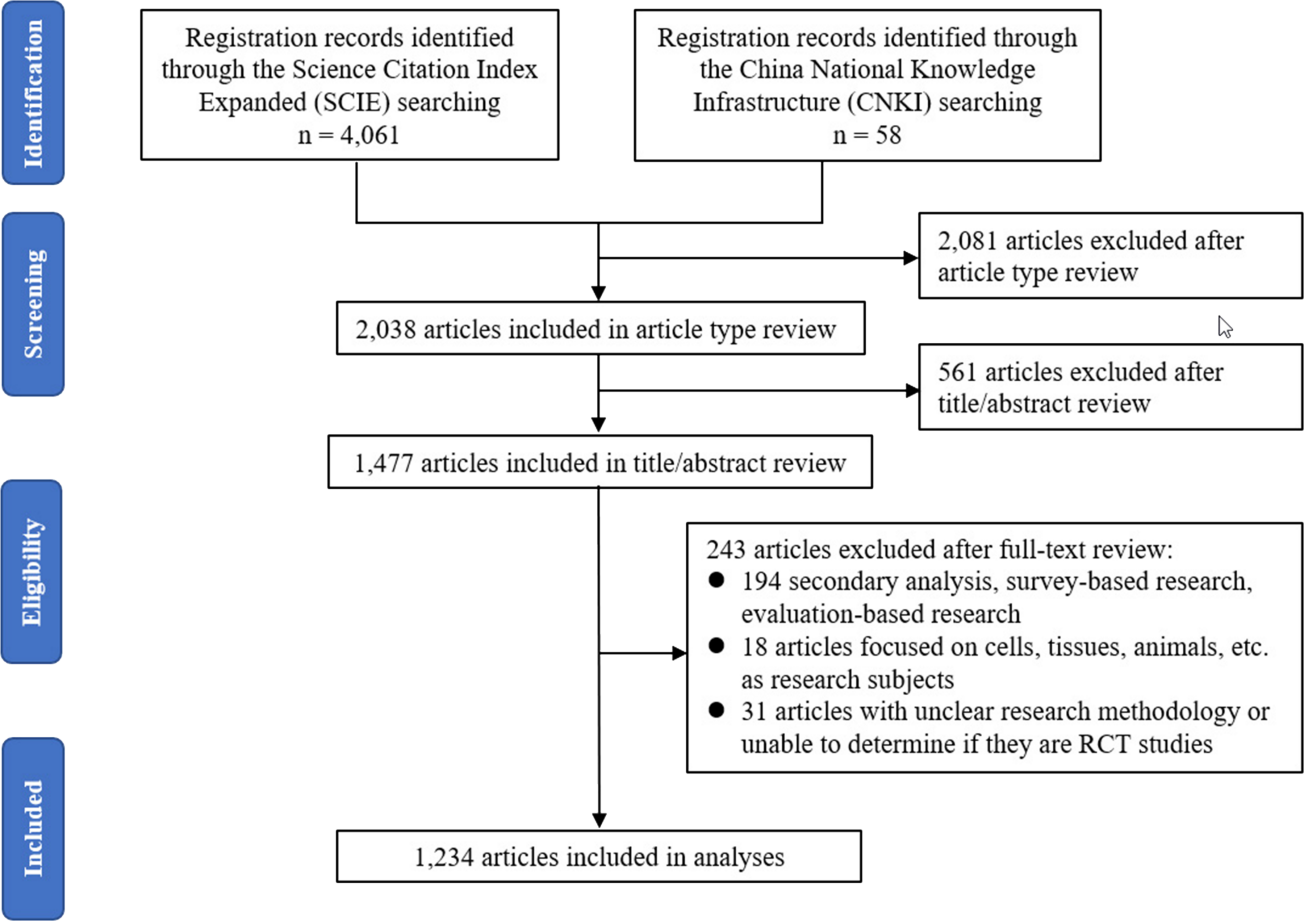
Flowchart of included articles.

Selection of RCTs. A total of 4,119 articles were initially identified through the literature search. After screening based on the predefined inclusion and exclusion criteria, 1,234 RCTs were included in the final analysis. The article selection process is shown in Fig. 1.

### Clinical trial registration

Among the 1,234 RCTs, 1,038 (84.1%) were registered as clinical trials. The registration rate for RCTs published in English-language journals was 85.0% (1,036/1,219), with 88.5% of these RCTs originating from journals that explicitly required clinical trial registration in their instructions for authors. In contrast, only 13.3% (2/15) of RCTs published in Chinese-language journals were registered, whereas these two journals that did not explicitly mandate clinical trial registration in their instructions for authors (Table 2).

**Table 2:** Clinical trial registration status of RCTs by different journal languages.

**Index**	**Total**	**Chinese-language journal**	**English-language journal**
RCTs	1,234	15	1,219
Registered RCTs	1,038/1,234 (84.1%)	2/15 (13.3%)	1,036/1,219 (85.0%)
Registered from required journals	917/1,038 (88.3%)	0/2 (0%)	917/1,036 (88.5%)

**Notes.**

RCT, randomized controlled trial.

Reporting of CONSORT flowcharts Overall, 83.5% (1,030/1,234) of RCTs reported a CONSORT flowchart. The proportion was higher in English-language journals (83.9%) than in Chinese-language journals (46.7%). Among RCTs that reported a CONSORT flowchart, approximately half were published in journals that explicitly endorsed CONSORT in their instructions for authors (Table 3).

**Table 3:** CONSORT flowchart publication status of RCTs by different journal language.

Index	Total	Chinese-language journal	English-language journal
RCTs	1,234	15	1,219
CONSORT flowcharts	1,030/1,234 (83.5%)	7/15 (46.7%)	1,023/1,219 (83.9%)
From CONSORT-required journals	550/1,030 (53.4%)	3/7 (42.9%)	547/1,023 (53.5%)

**Notes.**

RCT randomized controlled trial CONSORT Consolidated Standards of Reporting Trials

### Funding analysis

Among the 1,234 RCTs, 57.4% (708/1,234) reported receiving funding support. The proportion of RCTs with funding support in English-language journals was 60.5% (627/1,036) among those registered as clinical trials, whereas only 50.0% (1/2) of RCTS in Chinese-language journals reported receiving fund support. Among studies that received funding, 88.7% (628/708) were registered as clinical trials (Table 4).

**Table 4:** Analysis of funding support for RCTs.

**Index**	**RCTs in English-language journals (*n* = 1,219)**		**RCTs in Chinese-language journals (*n* = 15)**	
	Registered (*n* = 1,036)	Not registered (*n* = 183)	Registered (*n* = 2)	Not registered (*n* = 13)
Funded	627	70	1	10
Unfunded	409	113	1	3

**Notes.**

RCT, randomized controlled trial.

### Analysis of factors influencing clinical trial registration rates

Because only one Chinese-language journal adhered to the ICMJE recommendations, this analysis was restricted to the 51 English-language journals. As shown in Table 5, the clinical trial registration rate for RCTs published in ICMJE member journals was significantly higher than that in non-ICMJE member journals (90.4% *vs.* 82.1%). Similarly, the registration rate of RCTs in CONSORT member journals was significantly higher than that in non-CONSORT member journals (88.9% *vs.* 78.6%). Journals that explicitly required clinical trial registration in their instructions for authors have a significantly higher registration rate for RCTs than those that did not mention clinical trial registration (87.8% *vs.* 68.4%). Additionally, the registration rate for RCTs in journals that required adherence to the CONSORT statement was higher than that in journals that did not mention clinical trial registration (87.1% *vs.* 82.7%). Furthermore, RCTs with funding support had a significantly higher registration rate than those without (90.0% *vs.* 78.4%). These findings suggest that membership of the ICMJE and CONSORT, the inclusion of ICMJE recommendations and CONSORT statements in the instructions for authors, and fund support can enhance the registration rates of RCTs.

**Table 5:** Analysis of the factors affecting clinical trial registration rates in RCTs.

**Index**	**RCTs**	**Registered RCTs**	**Registration rate (%)**	***P* value**
ICMJE member journal				<0.001
Yes	426	385	385/426 (90.4%)	
No	793	651	651/793 (82.1%)	
CONSORT member journal				<0.001
Yes	757	673	673/757 (88.9%)	
No	462	363	363/462 (78.6%)	
Trial registration required				<0.001
Yes	1045	917	917/1,045 (87.8%)	
No	174	119	119/174 (68.4%)	
CONSORT adherence required				0.016
Yes	634	552	552/634 (87.1%)	
No	585	484	484/585 (82.7%)	
Funding support				<0.001
Yes	697	627	627/697 (90.0%)	
No	522	409	409/522 (78.4%)	

**Notes.**

RCT randomized controlled trial ICMJE International Committee of Medical Journal Editors CONSORT Consolidated Standards of Reporting Trials

Chi-square test was used to analyze factors associated with clinical trial registration rates, with *P* < 0.05 considered statistically significant.

### Analysis of registered clinical trial registries

Among the 1,038 RCTs that were registered as clinical trials, eight studies did not explicitly specify the registry. The remaining 1,030 RCTs were registered across 16 registries, with the top three registries being ClinicalTrials.gov (60.0% (623/1,030)), the Iranian Registry of Clinical Trials (IRCT) (11.5% (118/1,030)), and the Chinese Clinical Trial Registry (ChiCTR) (5.1% (53/1,030)) (Table 6).

**Table 6:** The number of registered RCTs among different registries.

**Registries**	**Registered RCTs**
ClinicalTrials.gov	623/1,030 (60.5%)
Iranian Registry of Clinical Trials (IRCT)	118/1,030 (11.5%)
Chinese Clinical Trial Registry (ChiCTR)	53/1,030 (5.1%)
International Standard Randomized Controlled Trial Number (ISRCTN)	45/1,030 (4.4%)
Australian New Zealand Clinical Trials Registry (ANZCTR)	42/1,030 (4.1%)
Netherlands National Trial Register (NTR)	29/1,030 (2.8%)
Clinical Trials Registry - India (CTRI)	25/1,030 (2.4%)
Pan African Clinical Trial Registry (PACTR)	19/1,030 (1.8%)
Brazilian Clinical Trials Registry (R.Bec)	17/1,030 (1.7%)
Thai Clinical Trials Registry (TCTR)	17/1,030 (1.7%)
EU Clinical Trials Register (EU-CTR)	16/1,030 (1.6%)
Japan Registry of Clinical Trials (jRCT)	12/1,030 (1.2%)
German Clinical Trials Register (DRKS)	7/1,030 (0.7%)
Clinical Research Information Service (CRIS), Republic of Korea	4/1,030 (0.4%)
Sri Lanka Clinical Trials Registry (SLCTR)	2/1,030 (0.2%)
Peruvian Clinical Trial Registry (REPEC)	1/1,030 (0.1%)

**Notes.**

RCT, randomized controlled trial.

**Figure 2:**
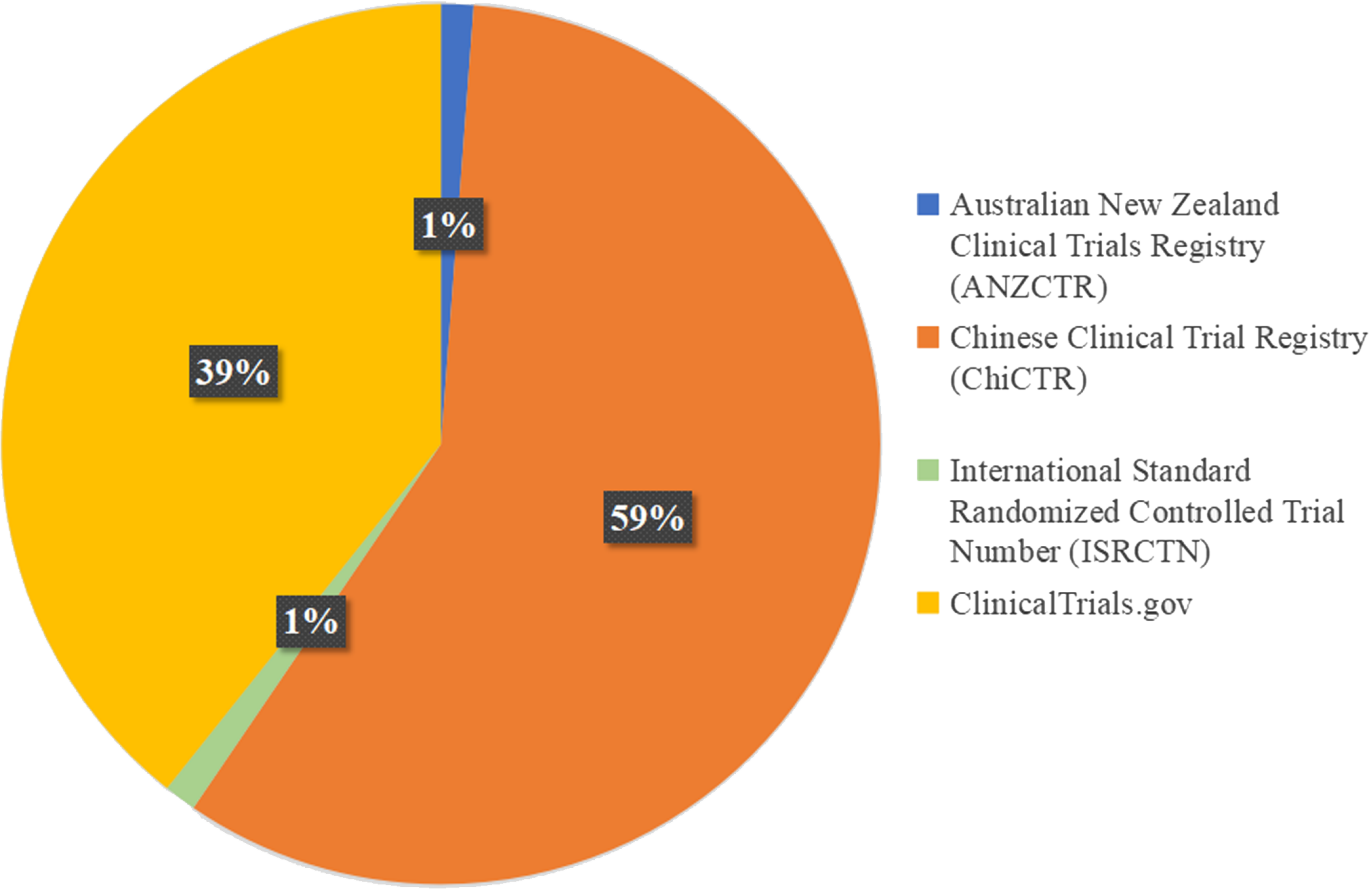
Registries for 84 RCTs published by Chinese scholars listed as corresponding authors.

Of the 1,038 RCTs, 86 were published by Chinese scholars listed as corresponding authors. Among these, one study provided a clinical trial registration number, but the corresponding registry could not be identified, and another article declared that it was registered but did not provide a registration number. After excluding these two articles, 84 RCTs published by Chinese corresponding authors were included in the analysis. Of these, 58.3% (49/84) were registered with the Chinese Clinical Trial Registry (ChiCTR), whereas 39.3% (33/84) were registered with ClinicalTrials.gov (Fig. 2).

### Analysis of the clinical trial registration rates by country

A total of 1,234 RCTs from 56 countries were analyzed. Among these, 29 countries had fewer than 10 RCTs, nine countries had 10–20 RCTs, and 18 countries had more than 30 RCTs. To minimize potential bias associated with small sample sizes, only countries with more than 30 RCTs were included. Sweden has the highest clinical trial registration rate (95.7%), followed by Denmark (95.5%) (Table 7). The clinical trial registration rate in China was 77.7%. Among the 97 RCTs published by Chinese scholars in English-language journals, 84 (86.6%) were registered, whereas only two out of 15 RCTs published in Chinese-language journals (13.3%) were registered.

**Table 7:** Clinical trial registration status of RCTs by country.

**Countries**	**Registered**	**Not registered**	**Registration rate (%)**
Sweden	22	1	22/23 (95.7%)
Denmark	21	1	21/22 (95.5%)
Australia	37	2	37/39 (94.9%)
Netherlands	34	3	34/37 (91.9%)
France	30	3	30/33 (90.9%)
Thailand	25	3	25/28 (89.3%)
Iran	123	16	123/139 (88.5%)
USA	209	28	209/237 (88.2%)
Brazil	33	5	33/38 (86.8%)
Israel	19	4	19/23 (82.6%)
UK	37	8	37/45 (82.2%)
Japan	19	5	19/24 (79.2%)
Egypt	69	19	69/88 (78.4%)
China	86	26	86/112 (77.7%)
Spain	31	10	31/41 (75.6%)
India	26	9	26/35 (74.3%)
Italy	19	10	19/29 (65.5%)
Turkey	35	19	35/54 (64.8%)

**Notes.**

RCT, randomized controlled trial.

## Discussion

In this cross-sectional analysis of obstetrics and gynecology journals, we examined journal policies related to clinical trial registration and adherence to the CONSORT statement, as well as the compliance with these requirements among published RCTs. By integrating journal-level requirements with article-level reporting behavior, our findings provided insight into how editorial policies may influence transparency and reporting practices within this specialty.

A key finding of this study is the substantial variability in journal policies regarding clinical trial registration and adherence to the CONSORT statement across publication contexts. English-language journals were more likely to require trial registration and to endorse the CONSORT statement than Chinese-language journals within this sample. Importantly, however, these differences should be interpreted as descriptive observations within a restricted set of obstetrics and gynecology journals, rather than as evidence of universal or national-level disparities. Our results highlight heterogeneity in editorial standards rather than definitive differences in research quality. Based on these findings, we further explored potential factors that may contribute to the observed discrepancies.

One possible explanation is that clinical trial registration and reporting standards have not yet been uniformly emphasized across Chinese medical journals. Among the eight Chinese-language journals included in this study, only one journal (*Chinese Journal of Obstetrics and Gynecology*) was a member of the ICMJE, and none were members of CONSORT. In contrast, among the 51 English-language journals surveyed, 16 were ICMJE members and 21 were CONSORT members. Furthermore, only one Chinese-language journal (*Chinese Journal of Reproductive and Contraception*) explicitly required RCTs to be registered and to adhere to the CONSORT statement in its instructions for authors. However, 36 of the 51 English-language journals required clinical trial registration, and 26 endorsed the CONSORT statement.

Growing evidence has demonstrated a strong association between journal policies and compliance with clinical trial registration and adherence to the CONSORT statement. Huser & Cimino (2013) analyzed 698 clinical trial articles published in five ICMJE founding journals, of which 95.8% had been registered. Sims et al. (2018) surveyed 300 RCTs published in the top 19 cardiovascular journals by impact factor in 2018 and found that 58% of the journals explicitly required clinical trial registration in their instructions for authors; 80.8% of the trials were registered, and 42.8% included CONSORT flowcharts. Wayant et al. (2018) investigated adherence to clinical trial registration policies in 21 oncology journals and found that 76.2% included such requirements in their instructions for authors. Of the 21 journals surveyed, 13 published RCTs, nine were ICMJE members, and 10 were CONSORT members. Among these trials, 67.4% were registered and 70.3% published CONSORT flowcharts. Cai et al. (2022) surveyed 131 Chinese biomedical journals and found that only 25.19% required clinical trial registration for RCTs in their instructions for authors, 27.48% required the reporting of the clinical trial registration number, and 21.37% required adherence to the CONSORT statement. Zhao et al. (2018) surveyed the registration status of RCTs published in the top 20 medical journals ranked by impact factors in the Chinese Science Citation Database (CSCD), revealing that only 4.8% of the RCTs were registered. Xu & Sun (2020) surveyed the registration status of prospective clinical trials in 20 high-impact-factor medical journals indexed in the CSCD, China, with only 7.4% of the trials indicating their registration institution and number. Li & Zhang (2019) surveyed 122 journals in the Chinese Medical Association series regarding the requirement for clinical trial registration numbers in their instructions for authors and found that 63.1% of the journals had such a requirement. Collectively, these findings clearly indicate a significant gap between Chinese- and English-language journals in terms of journal policies and clinical trial registration rates. Strengthening and clarifying such requirements in the instructions for authors may therefore represent an important step toward improving registration practices in Chinese-language journals.

From an editorial perspective, strict enforcement of clinical trial registration policies remains challenging. Wager & Williams (2013) surveyed 200 medical journals that published RCTs to understand their editorial policies and views on clinical trial registration. Among the 200 journals surveyed, 142 (71%) did not require clinical trial registration (or at least did not mention it on their journal homepage), 55 (28%) required clinical trial registration, and three (2%) encouraged clinical trial registration but did not require it for publication. Thirty-one journals with varying policies were further investigated, and several reasons were identified for not making clinical trial registration a prerequisite for publication: (1) a lack of clinical trial articles, (2) concern about being outcompeted by journals that do not require registration, (3) doubts about the effectiveness of clinical trial registration, and (4) concerns about limiting submissions from developing countries. Xu & Sun (2020) pointed out that mandatory implementation of clinical trial registration and publication mechanisms may affect submission rates, increase publication delays, and require additional time and effort from authors. Cai et al. (2022) also noted that if journal editors strictly enforce clinical trial registration, this could significantly reduce the number of articles passing the initial review, thereby decreasing their publication output. These practical considerations may partially explain why many journals have not yet adopted mandatory clinical trial registration policies.

In this study, 70.6% of English-language journals required RCTs to complete their clinical trial registration, whereas only 12.5% of Chinese-language journals included such requirements in their instruments for authors. Correspondingly, 85.0% of RCTs published in English-language journals had undergone clinical trial registration, compared with 13.3% of RCTs in Chinese-language journals. Within this sample of obstetrics and gynecology journals, Chinese-language journals showed lower reported rates of trial registration. Several factors may contribute to this pattern. First, variability in familiarity with internationally recognized editorial and ethical standards may affect the implementation of trial registration requirements. Editors of some journals may be uncertain about which types of clinical trials require registration and how registration policies should be operationalized. Second, competing editorial priorities may play a role. Under pressure to maintain publication volume, timeliness, and citation metrics, some journals may place less emphasis on enforcing trial registration requirements, thereby delaying the full implementation of registration systems. Third, differences in regulatory oversight and editorial review mechanisms may result in less standardized enforcement of trial registration policies.

In addition to journal-level factors, investigator-related considerations may also influence trial registration practices. Wu, Minawaer & Hao (2018) summarized the development of and issues with China’s clinical trial registration system over the past decade and found that the clinical registration rate in China was only 15%. One of the main reasons for this is that clinical professionals in China are not well-informed about the clinical trial registration system. Zhao et al. (2018) noted that clinical practitioners do not pay sufficient attention to the clinical trial registration system and lack knowledge of clinical trial methodology and implementation. Li & Zhang (2019) suggested that clinical researchers might be concerned about inadequate security measures that could lead to the leakage of trial protocols, results, and data, thus affecting their competitive advantage and making them reluctant to register for clinical trials. In addition, we believe that several other factors contribute to clinical practitioners not registering for clinical trials: (1) pending regulatory or ethical approval. Some clinical trials involve new drugs or technologies still in early development stages. Researchers may face prolonged regulatory reviews and approval processes, which make timely registration difficult; (2) publication pressure and bias. Some researchers may hesitate to register trials if the anticipate negative or inconclusive results, fearing subsequent publication difficulties. This is related to publication bias, the selective reporting of positive results while delaying or withholding negative results, which can lead to incomplete evidence and skewed clinical decision-making; (3) variations in registration platform requirements. Many countries and regions have established clinical trial registration platforms, with differing requirements and standards, particularly regarding data disclosure, which may create confusion or administrative burden for researchers. However, the submitted registration reports must comply with the requirements of the registration body. This may confuse some clinical medical practitioners, leading them to be relucatant to register their clinical trials.

A noteworthy finding of this study was that 86.60% of the RCTs published by Chinese scholars in English-language journals were registered, while only 13.33% of RCTs published in Chinese-language journals were registered. Zeng et al. (2018) assessed the reporting quality of RCTs on the treatment of hepatitis B and C published by Chinese scholars from 1991 to 2015. Among the 211 RCTs included in the study, 35.1% (74/211) were published in English-language journals and 64.9% (137/211) in Chinese-language journals. The proportion of RCTs in English-language journals that adhered to the CONSORT statement was 54.1%, whereas that in Chinese-language journals was only 46.8%. Their study demonstrated that the reporting quality of articles in the same field by the same researchers differs between Chinese- and English-language journals. From this perspective, this discrepancy cannot be simply attributed to a lack of knowledge or attention to the clinical trial registration system among Chinese clinical practitioners. Rather, it should be seen as an indication that Chinese-language medical journals have lower requirements for clinical trial registration and still publish unregistered trial reports. Li, Li & Liu (2005) analyzed the differences in the recognition and application of the CONSORT statement between domestic and international medical journals and called for improvements in the quality of both authors and editors to improve the quality of reporting in clinical trial and of medical journals.

### Strengths and limitations

This study has several strengths. First, it integrates journal-level policies with article-level reporting practices, providing a more comprehensive perspective on trial transparency than analyses focusing on either dimension alone. Second, the inclusion of both Chinese- and English-language journals allows for a comparative assessment across publication contexts within a single clinical specialty. Third, the use of standardized data extraction and independent review procedures enhances the reliability of the findings.

Several limitations should also be acknowledged. The number of Chinese-language journals included was relatively small compared with English-language journals, which limits direct comparability and may affect the precision of subgroup estimates. In addition, the study was restricted to obstetrics and gynecology journals, and the findings may not be generalizable to other medical specialties. Furthermore, adherence to the CONSORT statement was assessed using the presence of a CONSORT flowchart as a proxy indicator, which does not capture all dimensions of reporting quality. Finally, this study relied on publicly available information and did not assess the quality or completeness of registered trial records.

## Conclusion

Clinical trial registration represents a significant step toward fostering a more transparent clinical research environment. Since the establishment of the WHO ICTRP, the scientific community has accepted clinical trial registration and has become a key driver of global change in the research field. However, the current trial registration system has several shortcomings, and faces numerous challenges.

The findings of this study have practical implications for journal editors, reviewers, and authors within the field of obstetrics and gynecology. Clear editorial policies regarding trial registration and CONSORT adherence, coupled with consistent enforcement during peer review, may contribute to improved transparency and reporting practices. Encouraging the submission of CONSORT checklists and flowchart, as well as verification of trial registration at the time of manuscript submission, may represent feasible strategies to strengthen reporting standards.

## Supplemental Information

10.7717/peerj.21140/supp-1 Supplemental Information 1 Raw data

## Additional Information and Declarations

### Data Availability

The following information was supplied regarding data availability: The raw data is available in the Supplemental File.
